# After-School Behaviors, Self-Management, and Parental Involvement as Predictors of Academic Achievement in Adolescents

**DOI:** 10.3390/bs15020172

**Published:** 2025-02-06

**Authors:** Meng Xiao, Mingzhang Zuo, Xinqi Liu, Kunyu Wang, Heng Luo

**Affiliations:** Faculty of Artificial Intelligence in Education, Central China Normal University, Wuhan 430079, China; xm678@mails.ccnu.edu.cn (M.X.); liuxinqi0206@mails.ccnu.edu.cn (X.L.); ccnuwky2019@mails.ccnu.edu.cn (K.W.)

**Keywords:** after-school behaviors, self-management, parental involvement, academic achievement, adolescents, predictive factors

## Abstract

How adolescents’ after-school behaviors, self-management, and parental involvement are associated with their academic success and personal development has drawn growing attention. This study systematically examined how these three factors predict academic achievement. Data were collected from 353 seventh-grade students through a self-designed daily behavior log sheet and self-management app, and multiple linear regression analysis was conducted to identify key predictors. The results indicated that academic achievement was positively related to sleep quality, homework duration, self-assessment, and parental involvement, while it was negatively associated with sleep duration, exercise duration, and the number of daily planned tasks. Despite these insights, the study has limitations, including reliance on self-reported data, which may introduce bias, and the use of a cross-sectional design, which precludes determining the temporal sequence of variables and inferring causal relationships. These findings provide valuable insights for educators, parents, and students; they also emphasize the importance of organizing students’ after-school activities effectively, enhancing their self-management competencies, and encouraging active parental involvement in fostering students’ academic success and overall development.

## 1. Introduction

Adolescence is a critical period for academic and personal growth, and study habits and behaviors developed during this time have lasting effects. Academic achievement, a key indicator of learning outcomes, is closely linked to future academic and career success, as well as to psychological, emotional, and social development (M.-T. Wang & Eccles, 2013). Predicting academic performance is therefore quite important, because it empowers students to manage their learning and helps educators identify at-risk students, thus reducing academic failure rates (Bogarín et al., 2018). However, accurately predicting academic outcomes remains challenging due to the complex range of influencing factors, which are often categorized as individual, family, educational institution, and environmental elements (Hammouri, 2004). From the individual perspective, intelligence and learning strategies are strong predictors of academic success (Morales-Vives et al., 2020; Musso et al., 2020). Additionally, soft skills, such as adaptability, curiosity, and perseverance, also play a role in achievement through self-regulation and motivation (Feraco et al., 2023). From the perspectives of family, educational institutions, and the environment, student–teacher relationships directly predict grade point average (GPA), learning motivation, and perceived school climate, while parent–teacher relationships can indirectly predict GPA through motivation, especially in junior high school (Sethi & Scales, 2020).

Despite the importance of these factors, most existing research has focused on in-school influences, such as classroom performance and instructional quality (Alghamdi et al., 2020; Hattie, 2023). However, academic outcomes are shaped not only by what happens in school but also by a broad range of influences outside of school. After-school behaviors, such as extracurricular participation, social interactions, screen time, and physical activity, also contribute to academic outcomes in various ways, reflecting the multifaceted nature of adolescent development (Fredricks & Eccles, 2010; Saunders & Vallance, 2017). Recent studies have begun to examine the roles of students’ after-school behaviors, self-management, and parental involvement in academic outcomes (Boonk et al., 2018; Vandell et al., 2022; Zhu et al., 2020). However, existing research is often fragmented, focusing on isolated aspects rather than systematically analyzing how these factors collectively influence academic achievement. This lack of a comprehensive approach leaves a significant gap in understanding.

This study aims to address this gap by systematically examining the role of after-school behaviors (e.g., homework, exercise, and reading), self-management (e.g., sleep, self-assessment, and electronic device usage), and parental involvement in predicting academic achievement among adolescents. Utilizing a self-designed daily behavior log sheet and a self-management app, this study collected data on these factors and applied multiple linear regression models to assess their predictive power on academic achievement. Specifically, the study investigates the following research questions:

- What is the relationship between after-school behaviors and academic achievement in adolescents?
- How is self-management and academic performance related in adolescents?
- Is parental involvement associated with academic achievement in adolescents?

This study systematically analyzes these factors and contributes to a more comprehensive understanding of the complex predictors of academic performance, further expanding on the findings of previous studies. Additionally, the findings provide practical recommendations for educators, parents, and students to foster academic success and support personal growth during adolescence.

## 2. Related Work

### 2.1. After-School Behaviors

Examining students’ after-school behaviors is essential for fully understanding their academic development and personal growth. After-school behaviors can be broadly categorized into two types, namely school- or institution-organized activities and individualized activities. These categories reflect different dimensions of adolescent development, each contributing uniquely to students’ outcomes.

Many after-school activities in China are organized by schools or interest-based institutions, such as community centers, private tutoring organizations, or extracurricular training centers focused on music, arts, or sports. These activities usually take place in facilities provided by these organizations and are thus often viewed as extensions of the curriculum. For example, a student might attend piano lessons at a music academy, participate in basketball training at a local sports club, or engage in robotics workshops offered by a community center. Organized activities offer students diverse experiences and resources, allowing them to acquire valuable skills, build social connections, and develop self-efficacy—the belief in one’s ability to achieve goals and overcome challenges—and a positive sense of identity (Eccles et al., 2003; Feldman & Matjasko, 2005). Furthermore, research has demonstrated that students who participate more frequently in school-organized after-school activities tend to exhibit higher levels of learning motivation, more clearly defined academic goals, and improved academic performances (Fredricks & Eccles, 2010; Wolf et al., 2015). Beyond academic benefits, these activities also enhance social skills, mental well-being, and overall health (Agans et al., 2014; Lam & McHale, 2015).

While organized activities significantly contribute to student development, they are externally guided, and most existing research has focused on them. In contrast, individualized after-school behaviors are more likely to reflect students’ autonomy and self-management competencies, playing a critical role in shaping personality and contributing to long-term development (Larson, 2000; Mahoney et al., 2003). However, research on individualized after-school behaviors remains limited. This study aims to address this gap by exploring how these individualized behaviors contribute to academic performance.

Individual after-school behaviors considered in this study include completing homework, reviewing lessons, engaging in physical activities, and reading. These behaviors were selected because homework and review play crucial roles in learning by enhancing students’ understanding and retention of core concepts, helping them consolidate knowledge, and ultimately improving academic performance (Lee et al., 2022). Additionally, physical exercise and reading are also significant, as they contribute to cognitive development, learning abilities, social skills, and mental and physical well-being (Barbosa et al., 2020). Research has shown that students who regularly participate in physical exercise tend to achieve better academic results, while reading serves as a protective factor against academic failure and is positively linked to higher academic aspirations and performance (Barbosa et al., 2020; McGaha & Fitzpatrick, 2010). These findings highlight the multifaceted impacts of various after-school behaviors on adolescent development, offering a more comprehensive perspective on student growth during this crucial after-school period.

### 2.2. Self-Management

Self-management is the ability to enhance personal efficiency and achieve important goals through proactive goal setting, time planning, behavior monitoring, and outcome evaluation while demonstrating adaptability in the face of challenges (Agolla & Ongori, 2009; Al-Abyadh & Abdel Azeem, 2022). It also encompasses the ability to regulate thoughts, emotions, and behaviors across various situations to motivate oneself and strive toward academic and personal goals (Duckworth & Carlson, 2013).

Self-management competencies in students encompass four basic activities, including self-motivation, self-organization, self-control, and self-development. The first three foster and develop positive learning habits. In concrete terms, self-management encompasses abilities in time management, goal setting, self-monitoring, self-reflection and regulation, emotional control, resource management, and effort management. These competencies support students in maintaining focus, improving learning efficiency, overcoming challenges, and advancing personal growth (Jasim, 2020; Rahayu et al., 2024; Zhao et al., 2024).

Students’ self-management competencies are a key predictor of academic performance, decision-making, and behavioral correction (Deming, 2017; Stan, 2021). These competencies influence students’ capability and perseverance in achieving challenging goals, which makes them critical for realizing academic and personal aspirations (Bandura, 2001; Rahayu et al., 2024). Research has indicated that self-management has a direct and significant effect on improving classroom behavior, organizing learning activities effectively, enhancing homework and task completion and accuracy, and ultimately boosting academic performance (Smith et al., 2022; Stan, 2021; Zhao et al., 2024). Moreover, self-management indirectly enhances academic achievement by boosting students’ self-efficacy. Students with strong self-management competencies are more likely to develop higher self-efficacy, which then leads to improved performance. This finding aligns with Bandura’s (2001) theory that self-efficacy represents an individual’s subjective assessment of their capacity to successfully complete a task; this belief directly influences their behavior and performance (Al-Abyadh & Abdel Azeem, 2022).

Although self-management shares similarities with constructs such as self-regulation and executive functions (EFs), it is distinct in several ways. Self-regulated learning (SRL) is a broader theoretical framework that integrates metacognition, motivation, and behavioral regulation in the learning process. SRL emphasizes active planning, monitoring, and regulation of cognition, motivation, and behavior to achieve academic goals (Zimmerman, 2002; Pintrich, 2004). While self-management also involves monitoring and regulation, it is more practically oriented, focusing on competencies like time management, self-monitoring, goal setting, and emotional regulation to enhance personal efficiency and achieve important outcomes (Jasim, 2020; Zhao et al., 2024). Executive functions (EFs), on the other hand, refer to cognitive processes essential for goal-directed behavior, including inhibition, working memory, and cognitive flexibility (Diamond, 2013). While EFs are crucial for regulating thoughts and behaviors, self-management places a greater emphasis on the application of these cognitive skills in real-world contexts, such as managing tasks, setting goals, and regulating emotions in everyday life. In this study, we focus on self-management as a critical component of students’ ability to organize and regulate their behaviors to support academic success and personal development.

While self-management equips students with essential competencies for independent learning, these competencies are often nurtured and reinforced by external influences, particularly parental involvement. Parents play a pivotal role in shaping their children’s self-management capabilities through emotional support, guidance, and engagement in their educational activities, which will be explored in the next section.

### 2.3. Parental Involvement

Parental involvement plays a crucial role in adolescent academic development. It is generally recognized as a key predictor of academic success and refers to parental engagement in behaviors that support their children’s academic efforts (Barbosa et al., 2020). This engagement represents a comprehensive investment of material, time, and emotional resources in their children’s education (Barbosa et al., 2020; Yang et al., 2024). Parental involvement is often conceptualized as multidimensional, with varying definitions and measurements across studies. For example, Almroth et al. (2020) measured it by the frequency of parent–teacher meetings and attendance at school events, while H. Wang et al. (2023) categorized it into emotional, cognitive, and behavioral dimensions. Parental involvement generally takes three forms, including home-based involvement, school-based involvement, and parent–school communication (Chung et al., 2020; Hoover-Dempsey et al., 2005). Compared to the other two forms, home-based involvement emphasizes parent–child interactions. This type of involvement includes activities like homework assistance, exercising, reading together, and engaging in cognitive-stimulating outings, such as visits to zoos or museums. Understanding home-based involvement in the context of parenting styles is thus particularly important (Barbosa et al., 2020).

Numerous studies have confirmed the positive association between home-based parental involvement and various academic outcomes. For example, Gordon and Cui (2012) found that home-based involvement—such as helping with homework and projects, as well as discussing school life and academic progress—was significantly positively associated with adolescents’ GPA. Additional studies have explored the impact of parental involvement on specific academic subjects. Cui et al. (2021) observed that parents’ emotional and behavioral involvement positively influenced students’ math performance, while H. Wang et al. (2023) highlighted that parental involvement significantly predicted achievement in Chinese and English but not in mathematics. In addition, Sun et al. (2023) reported that parental emotional support and learning guidance positively affected academic outcomes, whereas family educational resources had a lesser influence. Factors that included family background, income level, and parent’s education level also moderated the relationship between parental involvement and academic achievement.

## 3. Methods

### 3.1. Participants

This study involved a sample of 372 seventh-grade students, aged 12–14, from 10 classes at a junior high school in Shenzhen, China. The selected school is representative of the region, with a student population drawn from various nearby communities and a diverse range of academic performance levels. The sample thus reflects the academic profile of local students in the target age group. The sample selection was randomized based on class lists provided by the school to ensure both randomness and representativeness.

During the data analysis phase, preliminary screening was conducted, which led to the exclusion of 19 students due to incomplete academic achievement data (e.g., resulting from missing exams or school transfers) or non-compliance with recording requirements for personal reasons. Following this screening, the final sample consisted of 353 students, including 188 boys and 165 girls, thus providing a reliable foundation for the study’s results.

### 3.2. Data Collection Instruments

Research has shown that self-assessment diaries serve as effective tools for cultivating self-management practices. These diaries involve participants regularly reflecting on and recording their thoughts, behaviors, and experiences over a period of time. This method encourages mindful self-monitoring, which supports behavioral change in a desired direction and enhances intrinsic motivation, self-efficacy, and academic performance (Klug et al., 2018; Yan, 2020). Accurate self-assessment is crucial in this process, as alignment between students’ self-assessments and external evaluations (e.g., from parents, teachers, or peers) significantly enhances the effectiveness of self-management strategies (Stan, 2021). Building on the strengths of self-assessment diaries, we developed two instruments to facilitate self-management practices, involving a paper-based daily behavior log sheet and a self-management app. The next sections detail the development and features of these instruments, which were designed to support students’ self-management and provide opportunities for reflection and feedback.

#### 3.2.1. Paper-Based Daily Behavior Log Sheet

We designed a paper-based daily behavior log sheet (see Appendix A) to better understand students’ after-school behaviors, self-management practices, and parental involvement. This customized instrument was developed to meet the unique requirements of our study, which existing tools were unable to fully address.

The design of this log sheet was primarily informed by theoretical research on self-management, self-regulation, and time management, as well as relevant educational guidelines, such as Canada’s 24-Hour Movement Guidelines for Children and Youth (Canadian Society for Exercise Physiology, 2016) and China’s Five-Point Management Directive (Ministry of Education of China, 2021). Drawing from our previous experiences with self-management projects and feedback from students, parents, and teachers, we identified key areas essential for students’ healthy development and self-growth. Based on these insights, we established the main activity categories for the log sheet, which include sleep, classroom learning, homework, review, reading, exercise, electronic device usage, self-assessment, and parental assessment. Most categories are assessed quantitatively using a 5-point Likert scale along with the time spent on each category. For example, the 1–5 Likert scale for active participation in Chinese classroom learning ranges from 1 (inactive) to 5 (very active). Similarly, the scale for concentration in homework ranges from 1 (very distracted) to 5 (very focused).

#### 3.2.2. Self-Management App

We developed a self-management app (see Appendix B) based on the paper-based daily behavior log sheet to streamline data collection and expand its functionality. The app replicates the activities included in the paper log sheet and incorporates advanced features to provide detailed feedback on students’ after-school behaviors and self-management performance. It generates real-time feedback reports for daily, weekly, or custom periods, enabling students to monitor their status and track their progress at any time.

To help junior high school students limit their use of electronic devices, they first complete the paper log sheet by recording their daily activities. This approach reduces the need for prolonged screen exposure while still allowing for data tracking. Parents can then use the app’s optical character recognition (OCR) function to upload a photo of the completed log sheet. This feature achieves over 95% accuracy, ensuring high data quality and seamless integration of data into the app. Students could then access downloadable feedback reports, compare their performance to previous records and class averages, and engage in self-monitoring and reflection to promote effective self-regulation.

### 3.3. Research Procedure

This study was conducted from October 2022 to May 2023. Throughout the study, the research team adhered strictly to data privacy and ethical standards to ensure that all of the participants’ personal information was properly managed and protected. Informed consent was obtained from both the participants and their parents or guardians, which confirmed that participation was entirely voluntary and that they could withdraw at any time. The study protocol was approved by the Ethics Committee of Central China Normal University (CCNU-IRB-202104016b).

The preparation phase (17 to 23 October 2022) involved the research team conducting multiple project orientation and Q&A sessions for students, parents, and teachers. These sessions aimed to ensure that participants and their guardians fully understood the study’s purpose and content. Key topics included the significance of students’ after-school behaviors, self-management, and parental involvement. The sessions also provided details on project implementation, guidelines for completing the paper-based daily behavior log sheet, and instructions for using the self-management app, with particular emphasis on the optical character recognition (OCR) function. An electronic version of the app’s operation manual was distributed to participants for reference. Following these sessions, a one-week pretest was conducted to assess the usability and functionality of both the paper-based log sheet and the self-management app. Participating students, parents, and teachers were invited to use the tools and provide feedback on the clarity, usability, and relevance. This feedback allowed the team to make timely adjustments, optimizing the tools for smooth implementation and effective data collection during the main study.

The data collection phase (24 October to 19 December 2022) focused on students’ completion of the paper-based behavior log sheet daily. Parents were responsible for uploading the completed log sheet data through the self-management app in order to regulate students’ screen time. The research team periodically sampled and verified a portion of the data to ensure accuracy. Any anomalies (e.g., excessive missing data or inconsistencies) were promptly addressed through communication with the students and parents. The research team also collected students’ academic performance data from their final exams for the first semester of seventh grade in December 2022. The students’ mid-term exam scores from the second semester of seventh grade were also collected in May 2023 in order to further explore the lasting impact of the project. These data covering academic performance provided crucial information for subsequent research analyses.

### 3.4. Data Analysis

This study identified the following 15 potential variables from the daily behavior log sheet that may be associated with students’ academic performance: sleep duration, sleep quality, total number of classroom learning reflections, homework duration, review duration, reading duration, exercise duration, duration of electronic device use, daily mood, self-assessment scores, parental assessment scores, parental involvement scores, number of daily planned tasks, daily task execution rate, and total days of log sheet recording. These variables were selected based on theories of self-management, self-regulation, and time management, as well as previous research linking student behavior to academic outcomes. Data analysis was conducted using SPSS 26.0, employing the backward elimination method in multiple regression analysis. This approach was chosen to account for the potential interactions between multiple independent variables. It allowed for a comprehensive examination of the relationships and minimized the risk of prematurely discarding significant factors.

Due to the voluntary nature of student participation in the self-management project, the heavy academic workload of seventh-grade students, and the challenges posed by the COVID-19 pandemic, some students occasionally forgot to complete their log sheets. From October to November 2022, students were required to comply with school and community COVID-19 prevention measures, which disrupted their daily routines. In December 2022, after China lifted all COVID-19 restrictions, some students and their families contracted the virus, further affecting their participation. To ensure data consistency, the study averaged the daily behavior log data over two months for all students. While averaging may slightly alter the relationships between variables, it helps normalize the data distribution, thus facilitating subsequent analysis.

Given the time-consuming and complex nature of processing two months’ worth of daily behavior log data from 353 students, mean imputation was chosen as a practical and efficient method for handling missing data. This choice was driven by the need to balance analytical rigor with the constraints of time and resources. While we acknowledge that alternative methods may offer certain advantages, mean imputation was deemed the most appropriate for this study, ensuring data completeness while maintaining analytical feasibility. To evaluate the potential bias introduced by mean imputation, we compared the characteristics of the data and the results of the analysis before and after imputation. No significant differences were observed, indicating that the imputation did not distort the key relationships among the study’s primary variables. Furthermore, the missing data were found to follow a random distribution pattern, which further justified the use of mean imputation in this study.

## 4. Results

Table 1 contains descriptive statistics for the demographic variables in this study.

Table 2 presents the Pearson correlation coefficients among the study variables. It shows the relationships between various factors (such as sleep duration, self-assessment, parental involvement, etc.) and academic achievement (measured at two different time points, *y*1 for the first semester and *y*2 for the second semester).

The results indicate significant positive correlations between sleep quality (*x*2), total number of classroom reflections (*x*3), homework duration *(x*4), self-assessment scores (*x*10), parental assessment scores (*x*11), parental involvement scores (*x*12), and the total days of log sheet recording (*x*13) with academic performance at both time points (*y*1 and *y*2). These variables appear to have a beneficial impact on academic achievement. In contrast, sleep duration (*x*1) was found to have a significantly negative correlation with academic performance at both time points (*y*1 and *y*2), suggesting that longer sleep duration may not contribute positively to students’ academic performance. The daily task execution rate (*x*15) showed a significant positive correlation with student achievement only in the second semester (*y*2), suggesting that task execution might become more critical over time. Several variables, such as the total number of classroom learning reflections (*x*3), parental involvement scores (*x*12), and total days of log sheet recording (*x*13), showed high correlations with each other. These high correlations may indicate potential multicollinearity issues, which should be taken into account in subsequent regression analyses.

The backward elimination method in multiple linear regression was employed to sequentially remove non-significant variables (*p* > 0.05) from the full model, resulting in the most parsimonious model. The final models included only variables with *p*-values below 0.05, confirming their statistical significance. The results indicated key predictors of students’ academic performance; seven variables were significantly associated with the final exam scores of the seventh-grade students in the first semester (see Table 3) and six variables were significantly related to the midterm scores in the second semester (see Table 4). Notably, sleep duration, homework duration, and self-assessment scores consistently emerged as the strongest predictors across both exams. For comprehensive results, including the variables removed during the backward elimination process, refer to Table A1 and Table A2 in Appendix C.

As shown in Table 3, the seven variables significantly associated with students’ final exam scores in the first semester of seventh grade were sleep duration, sleep quality, homework duration, exercise duration, self-assessment scores, parental involvement scores, and the number of daily planned tasks. Among these, sleep quality, homework duration, self-assessment scores, and parental involvement scores were positively related to academic performance, suggesting that quality sleep, appropriate homework practices, high self-assessment scores, and active parental involvement contribute to better academic outcomes. Conversely, sleep duration, exercise duration, and the number of daily planned tasks were negatively related to scores, indicating that excessive sleep and exercise, as well as too many planned tasks, may hinder academic performance.

Table 4 indicates that six variables—sleep duration, sleep quality, homework duration, self-assessment scores, parental involvement scores, and the number of daily planned tasks—were significantly associated with midterm scores for the second semester of seventh grade. Compared to the first semester (see Table 3), exercise duration no longer had a significant effect. The positive relationships of sleep quality, homework duration, self-assessment scores, and parental involvement scores with academic performance remained consistent, while sleep duration and the number of daily planned tasks continued to show negative relationships.

The significant variables associated with both exams appeared to remain largely consistent. Sleep quality, homework duration, self-assessment scores, and parental involvement scores were positively related to students’ academic performance in both semesters. In contrast, sleep duration and the number of daily planned tasks were negatively related to scores. Interestingly, the negative relationship between exercise duration and academic performance observed in the first semester disappeared in the second semester, which differs from some research on exercise and academic performance. This discrepancy may be due to differences in how exercise duration was measured or the changing circumstances students encountered each semester. Additionally, in contrast to findings from previous studies, the duration of electronic device use showed no significant association with academic performance in either semester. These differences suggest that further investigation is needed.

## 5. Discussion

This study examined the complex factors influencing adolescents’ academic achievement, which encompass personal behaviors (e.g., homework duration and exercise duration), intrapersonal variables (e.g., self-assessment and daily mood), interpersonal relationships (e.g., the impact of parental involvement), and environmental influences (e.g., the lasting effects of good habits and environments). Within this broader context, the study specifically focused on the role of after-school behaviors, self-management, and parental involvement and explores how these elements contribute to academic performance.

Using multiple linear regression analysis, this research investigated the complex relationship between daily indicators and academic performance among 353 seventh-grade students. The analysis identified key predictors significantly associated with academic achievement. Specifically, sleep quality, homework duration, self-assessment scores, and parental involvement scores were positively related to academic performance. In contrast, sleep duration, exercise duration, and the number of daily planned tasks were negatively related to academic achievement. Notably, the duration of electronic device use did not show a significant association with academic outcomes. These findings underscore the multifaceted nature of academic performance and highlight the importance of both individual behaviors and external influences in shaping students’ academic success.

The next sections discuss these key predictors in detail and explore how factors like homework duration, exercise duration, sleep duration, self-assessment scores, duration of electronic device use, and parental involvement scores are related to academic outcomes, with recommendations for educational practices.

### 5.1. Key Predictors

#### 5.1.1. Homework Duration

The positive relationship between homework duration and academic achievement has been well-documented in numerous studies (H. Cooper et al., 2006; Trautwein, 2007). Students who spend more time on homework tend to achieve higher academic outcomes. The present study also found a significant positive association between homework duration and students’ academic performance. Allocating more time to homework helps students review and consolidate knowledge, identify gaps in their understanding, and deepen comprehension, ultimately leading to better exam results.

A homework duration of 1–2 h per night is most strongly associated with academic improvement for junior high school students (H. M. Cooper, 1989). However, exceeding this range does not necessarily lead to continued gains and may even result in ineffective study habits or counterproductive routines (H. M. Cooper, 1989; Trautwein & Köller, 2003). Students with lower academic performance or less prior knowledge may also require more time to complete homework (Trautwein, 2007).

Notably, the association between homework duration and academic performance is more pronounced when self-reported by students rather than by parents (H. Cooper et al., 2006). The use of a student-completed daily behavior log sheet in this study thus provided a more accurate measure of the actual time spent on homework.

#### 5.1.2. Exercise Duration

Numerous studies have demonstrated the positive effects of physical exercise on adolescents’ cognitive function, academic outcomes, and mental health (Marques et al., 2017; Poitras et al., 2016; WHO, 2020). For instance, Du et al. (2023) found that students engaging in moderate exercise, such as running once per week for 16–25 min, performed better academically than approximately 88% of their peers. However, contrary to these findings, the present study identified a negative association between time spent on physical activity and academic achievement.

Several factors may explain this unexpected result. First, the heavy academic workload faced by junior high school students in our sample may play a significant role. Prolonged physical activity, while beneficial to overall health, could lead to fatigue and reduce the time and energy available for studying, thereby diminishing learning efficiency. This is in line with Lindner (2002), who observed no positive association between academic performance and physical activity, with students in secondary grades 4 and 6 showing no significant relationship and students in secondary grade 2 showing a slight negative association. Second, the intensity and type of physical exercise may play a role. Esteban-Cornejo et al. (2014) also found a negative association between time spent at different intensities of physical activity and academic performance when overall physical activity was not considered. Since our study did not differentiate between types or intensities of exercise, this limitation could partially explain the observed negative association.

Third, cultural and contextual factors within educational systems may have contributed to these findings. In some countries, concerns that non-academic subjects, such as physical education, may hinder academic outcomes have led some schools to reduce time allocated to physical activities (Lindner, 2002; Marques et al., 2017; UNESCO, 2014). Adolescents who engage in more physical activities often dedicate less time to studying, potentially impacting their focus and learning efficiency, thus potentially affecting academic performance.

Future efforts should help students find a balance between physical exercise and academics, ensuring that students benefit from exercise without compromising their academic outcomes.

#### 5.1.3. Sleep Duration

Sleep is essential for learning, memory consolidation, and attention among adolescents, as these factors are related to academic performance and long-term development (Gómez-Fonseca & Genzel, 2020; Musshafen et al., 2021). Although previous research has generally shown a positive association between sleep duration and academic achievement (Adelantado-Renau et al., 2019), our study unexpectedly found a negative association.

One possible explanation is that excessive sleep duration, beyond the recommended 8–10 h, may reduce the time available for studying and decrease engagement in academic activities. This aligns with findings by Chaput et al. (2016), who reported that adolescents sleeping far beyond the normal range may face health issues that impair academic outcomes. Additionally, Adelantado-Renau et al. (2019) found no association between self-reported sleep duration and academic outcomes. Such discrepancies may stem from differences in measurement methods. Subjective self-reports often overestimate actual sleep time compared to objective tools. For example, the time spent in bed is typically longer than actual sleep time, which can result in overreporting (Hirshkowitz et al., 2015). Our discussions with students also revealed varying sleep needs based on individual conditions, which could influence responses to sleep duration. Overall, we found that while appropriate sleep duration is generally linked to better academic performance, deviations from the recommended 8–10 h are related to poorer outcomes (Faught et al., 2017).

Future research should consider multiple dimensions of sleep, such as quality, efficiency, and individual variability in sleep needs, while employing objective measurement tools to better understand the nuanced relationship between sleep and academic performance.

#### 5.1.4. Self-Assessment Scores

Self-assessment is a key predictor reflecting students’ self-awareness and confidence; it has been closely linked to academic performance, and this relationship has been consistently confirmed in multiple studies (Martínez et al., 2020; Yan et al., 2020). Our findings further support this and showed that engaging in self-assessment significantly enhanced academic achievement. Regular self-assessment is an effective strategy that fosters reflection, autonomy, and engagement in learning, thus leading to improved behaviors and higher achievement (Martínez et al., 2020). Lishinski and Yadav (2021) found that students who completed self-assessments scored significantly higher on projects than those who did not. Self-assessment allows students to identify needs, set goals, and create action plans to achieve those goals, thereby boosting motivation and confidence. It also cultivates critical thinking and analytical skills, thus guiding students toward self-regulated learning (Martínez et al., 2020).

#### 5.1.5. Duration of Electronic Device Use

Electronic devices play a central role in the daily life of adolescents. These devices facilitate learning, entertainment, and social interaction. However, contrary to some previous studies, we found that the time spent on electronic devices was not a significant predictor of academic achievement. While many studies have suggested that increased device usage may be associated with dependency and negatively related to learning outcomes (Saunders & Vallance, 2017; Winskel et al., 2019), this relationship is not universally supported. First, simply measuring screen time may not fully capture how students use these electronic devices (Athavale et al., 2021). Second, the emotional support, entertainment, and social interactions derived from electronic device use (David et al., 2015) may offset potential negative effects, which makes assessing the impact on academic achievement more complex.

Our communication with students and parents also revealed individual differences in screen time regulation. Some students effectively managed their screen time, ensuring it did not interfere with their academics, while others became overly absorbed, thus negatively affecting their studies. This variability may explain the lack of significant association between electronic device use and academic performance in our analysis. Future research on this relationship should consider examining multiple behavioral variables related to electronic device use rather than focusing solely on screen time.

#### 5.1.6. Parental Involvement Scores

This study also confirmed the positive association between parental involvement and students’ academic success, which is consistent with previous research showing that students generally perform better with parental support (Patall et al., 2008). Parental involvement is linked not only to academic performance but also to children’s overall development and educational attainment. Studies have consistently demonstrated that higher levels of parental involvement are associated with greater potential achievements in children (Schmid & Garrels, 2021; Sujarwo & Herwin, 2023). Wilder (2023) further noted that regardless of how parental involvement is defined or how achievement is measured, this positive relationship holds across different grade levels and ethnic groups. However, while parental involvement typically benefits academic outcomes, the effect is moderate. If it creates pressure or undermines the child’s autonomy, it may negatively affect achievement. Conversely, supportive and autonomy-promoting involvement tends to be positively related to success (Moroni et al., 2015). In this study, parental involvement was measured by the frequency of ratings and comments in the daily behavior log sheet, without assessing quality. Future research could explore the quality of parental involvement to better understand its effects.

### 5.2. Lasting Effect of Habitus

This study analyzed the final exam scores of seventh-grade students in the first semester and the midterm exam scores in the second semester. The aim was to explore whether the behavior habits formed by students in the first semester through filling out a daily behavior log sheet could predict their academic performance in the second semester, when they no longer completed the log sheet. The data analysis results indicate that the behavior habits developed by students in the first semester have a lasting and significant impact on their academic performance in the second semester. The indicators used to predict academic performance were essentially consistent across both time points. It is important to note that the behavior habits recorded by students in the first semester were not directly cultivated by the log sheet itself. The purpose of students filling out the daily behavior log sheet was to help them pay more attention to and reflect on their existing behavior habits.

This study is informed by Bourdieu’s (1993) field theory, which posits that both school and family constitute the educational field that influences student development. In this field, students engage in the process of monitoring and reflecting on their daily behavior by filling out the behavior log sheet, a process that is closely linked to the resources and support provided by both schools and families. The interaction between these two fields shapes students’ behavior patterns, gradually internalizing them into students’ “habitus”.

Within the school field, teachers guide students in filling out the behavior log sheet, helping them monitor and reflect on their behavior while simultaneously transmitting cultural capital (such as learning strategies, knowledge, and skills) (Gaddis, 2013). This cultural capital is gradually internalized through the students’ learning processes, influencing their learning habits and academic performance. In the family field, parents remind students to complete their daily behavior log sheets, review the feedback reports downloaded from the app with them, compare the reports with the student’s previous performance and the class average, and participate in shaping the students’ learning behavior norms. Additionally, a family’s economic and social capital can influence students’ behavior. For example, families with better economic conditions can provide more learning resources, while families with extensive social networks may offer access to broader learning perspectives, which could affect students’ log sheet content and self-evaluations.

Through the interactive influence of the school and family fields, students gradually form specific behavior patterns, which Bourdieu refers to as “habitus.” For instance, students may develop the habit of recording their learning activities, after-school behaviors, and self-management practices daily, encouraging attention to time management and personal well-being. Over time, these behavior patterns are internalized into students’ subconscious tendencies, such as conscious task planning and self-reflection, further consolidating their habitus (Bourdieu, 1977).

This study finds that daily behavior log sheets help students enhance their self-monitoring and self-regulation abilities, foster the formation of positive habits through behavioral monitoring and reflection, and have a lasting impact on academic performance. Although the behavior log sheets in the first semester primarily aid students in recognizing and reflecting on their existing behavior habits, this process lays a foundation for their subsequent learning and personal development. This finding underscores the importance of fostering good behavior habits in educational practice.

### 5.3. Implications

This study provides valuable insights for educators, parents, and students, emphasizing the importance of optimizing after-school behaviors, self-management, and parental involvement to enhance academic performance and holistic development in adolescents. These findings align with and extend existing research, offering actionable recommendations for key stakeholders.

For educators, the findings emphasize the importance of assigning reasonable homework loads to ensure optimal homework duration. Guiding students to establish balanced schedules and develop self-management competencies—such as goal setting, task execution, self-assessment, and reflection—is crucial, as self-regulation is strongly linked to academic success (Zimmerman, 2002). Additionally, promoting moderate physical activity and providing timely constructive feedback can enhance student motivation and foster a supportive learning environment (Hattie & Timperley, 2007).

For parents, the study highlights the necessity of creating a comfortable and supportive environment for sleeping and studying, as these factors play a critical role in academic performance. Encouraging balanced after-school activities, such as moderate physical exercise, aligns with research demonstrating benefits for both physical and mental health (Barbosa et al., 2020). Moreover, helping children establish effective study routines, fostering reflective habits, and providing consistent positive feedback are well-supported strategies for enhancing academic growth.

For students, ensuring sufficient and high-quality sleep is foundational, as it supports cognitive functioning, memory consolidation, and sustained attention (Hirshkowitz et al., 2015). Regularly planning tasks, diligently completing homework, effectively managing after-school activities, balancing study and leisure, and cultivating consistent and moderate exercise routines are integral to academic success. Furthermore, developing self-management competencies—such as time management and goal setting—not only enhances academic performance but also helps establish sustainable habits for long-term personal and academic growth.

### 5.4. Limitations and Future Research

Although this study provides new perspectives and empirical support for the factors associated with adolescents’ academic performance, it has several limitations.

First, the study relied on a self-designed daily behavior log sheet and self-management app. Although these tools were innovative, they have not undergone extensive validation across diverse educational contexts. For instance, variables such as electronic device usage were not specific enough to distinguish between time spent on learning and entertainment, limiting the nuanced understanding of how device usage impacts academic performance. Furthermore, the variable settings for after-school behavior in the log sheet are somewhat limited, failing to capture the full range of activities students engage in after school, restricting a holistic assessment of their after-school lives and their interplay with academic outcomes. Future research should prioritize refining and validating these instruments across broader populations while incorporating more comprehensive activity categories to enhance precision and applicability.

Second, self-reports were the primary data source. While self-reporting is a widely used method in research on self-efficacy and personal development, it is prone to biases such as recall inaccuracies and social desirability effects, potentially compromising data quality (Paulhus & Vazire, 2007). Future studies should consider integrating objective measures, such as digital activity trackers, observational techniques, or teacher ratings, to complement self-reported data and improve validity.

Third, the research sample consisted of seventh-grade students from a junior high school in Shenzhen, a highly economically developed city in China. To protect the privacy of students and their families, data on family income and other socioeconomic indicators were not collected. This limitation prevented an analysis of how economic disparities might influence students’ participation in extracurricular activities or the level of parental support they receive. Future research should include a broader range of socioeconomic backgrounds to enhance the generalizability of the findings and provide a more comprehensive understanding of these dynamics.

Fourth, the cross-sectional design of this study limits its ability to establish causal relationships or determine the temporal sequencing of variables. Future research is recommended to adopt longitudinal designs to investigate the dynamic and reciprocal interactions among after-school behaviors, self-management, and academic performance over time.

Finally, the COVID-19 pandemic posed significant challenges to data collection and student participation. School closures, preventive measures, and illness disrupted students’ routines during the study period. To address these disruptions, daily behavior log data were averaged over two months, and missing values were managed using mean imputation. While these approaches helped standardize the dataset, they may have influenced the relationships among variables. Future research should adopt more robust methods, such as sensitivity analyses or alternative imputation techniques, to account for disruptions and assess their potential impacts comprehensively.

By addressing these limitations, future studies can further elucidate the complex relationships among after-school behaviors, self-management, parental involvement, and academic achievement, providing actionable insights for educators, parents, and policymakers. Future research should investigate long-term effects, diverse educational settings, and cross-cultural contexts. Moreover, expanding sample diversity, integrating multiple data collection methods, and refining measurement tools could strengthen the robustness of findings, offering more effective guidance for improving students’ academic outcomes and supporting their personal development.

## 6. Conclusions

This study used a self-designed daily behavior log sheet and self-management app to collect data on junior high school students’ after-school behaviors, self-management, and parental involvement. We examined the associations between these variables and adolescent academic achievement. The results revealed that academic achievement was positively related to sleep quality, homework duration, self-assessment, and parental involvement, while it was negatively associated with sleep duration, exercise duration, and the number of daily planned tasks.

These findings highlight the complex multifaceted nature of academic performance, where multiple individual, interpersonal, and environmental factors interact to influence students’ outcomes. Specifically, our study emphasizes the importance of after-school behaviors, self-management, and parental involvement in shaping academic success. The results contribute to a deeper understanding of how these factors, often studied in isolation, work together to support academic achievement. Moreover, this study underscores the shared responsibility of educators, parents, and students in fostering academic success and personal development. By guiding students in structuring their after-school activities, enhancing self-management skills, and providing supportive parental involvement, all parties can contribute to students’ holistic development. This research adds to the growing body of the literature on the complex interactions that influence academic performance, providing valuable insights for educational practice and future research.

## Figures and Tables

**Table 1:** Survey participants’ descriptive statistics.

Variable		Frequency %		Mean ± SD	Minimum	Maximum
Age		—	—	12.39 ± 0.50	12	14
Sex	Male	188	53.3	—	—	—
	Female	165	46.7	—	—	—

**Table 2:** Correlation matrix of all variables.

	*y*1	*y*2	*x*1	*x*2	*x*3	*x*4	*x*5	*x*6	*x*7	*x*8	*x*9	*x*10	*x*11	*x*12	*x*13	*x*14	*x*15
*y*1	1																
*y*2	0.943 **	1															
*x*1	−0.172 **	−0.162 **	1														
*x*2	0.122 *	0.111 *	0.199 **	1													
*x*3	0.203 **	0.184 **	0.01	0.108 *	1												
*x*4	0.183 **	0.186 **	0.028	−0.001	0.086	1											
*x*5	0.06	0.062	0.024	0.186 **	0.052	0.311 **	1										
*x*6	−0.09	−0.069	0.023	0.073	−0.112 *	0.075	0.109 *	1									
*x*7	0.05	0.063	0.138 **	0.246 **	0.079	0.168 **	0.166 **	0.154 **	1								
*x*8	−0.073	−0.07	0.148 **	−0.026	−0.140 **	0.035	0.015	0.138 **	0.134 *	1							
*x*9	0.068	0.068	0.221 **	0.374 **	0.04	0.021	0.149 **	0.102	0.230 **	0.117 *	1						
*x*10	0.156 **	0.157 **	0.106 *	0.260 **	0.056	−0.018	0.098	0.107 *	0.176 **	0.128 *	0.739 **	1					
*x*11	0.160 **	0.164 **	0.1	0.249 **	0.064	0.148 **	0.158 **	−0.02	0.197 **	0.078	0.467 **	0.540 **	1				
*x*12	0.207 **	0.184 **	0.04	0.128 *	0.870 **	0.114 *	0.086	−0.094	0.086	−0.116 *	0.063	0.058	0.085	1			
*x*13	0.188 **	0.172 **	0.046	0.099	.947 **	0.124 *	0.068	−0.039	0.063	−0.133 *	0.036	0.046	0.055	0.865 **	1		
*x*14	−0.075	−0.093	0.171 **	0.222 **	−0.106 *	0.139 **	0.197 **	0.023	0.418 **	0.024	0.296 **	0.168 **	0.221 **	−0.05	−0.111 *	1	
*x*15	0.098	0.114 *	0.132 *	0.154 **	0.133 *	0.202 **	0.124 *	0.028	0.129 *	0.02	0.237 **	0.276 **	0.158 **	0.125 *	0.124 *	0.041	1

Pearson correlation coefficients are presented. Sample size = 353. ** Significant at *p* < 0.01. * Significant at *p* < 0.05. *y*1: end of the first semester in grade 7; *y*2: middle of the second semester in grade 7; *x*1: sleep duration (h); *x*2: sleep quality; *x*3: total number of classroom learning reflections; *x*4: homework duration (min); *x*5: review duration (min); *x*6: exercise duration (min); *x*7: reading duration (min); *x*8: duration of electronic device use (min); *x*9: daily mood scores; *x*10: self-assessment scores; *x*11: parental assessment scores; *x*12: parental involvement scores; *x*13: total days of log sheet recording; *x*14: number of daily planned tasks; *x*15: daily task execution rate.

**Table 3:** Indicators at the end of the first semester of grade 7.

Variable	*B*	SE	*t*	*P*	VIF
Sleep duration (h)	−0.206	0.051	−4.055 **	0.000	1.062
Sleep quality	0.134	0.053	2.519 *	0.012	1.164
Homework duration (min)	0.200	0.050	3.959 **	0.000	1.049
Exercise duration (min)	−0.112	0.050	−2.227 *	0.027	1.035
Self-assessment scores	0.169	0.052	3.259 **	0.001	1.104
Parental involvement scores	0.149	0.051	2.941 **	0.003	1.058
Number of daily planned tasks	−0.115	0.052	−2.210 *	0.028	1.120
Duration of electronic device use (min)	−0.070	0.052	−1.343	0.180	1.130

Note: SE = standard error; VIF = variance inflation factor. ** Significant at *p* < 0.01. * Significant at *p* < 0.05.

**Table 4:** Indicators in the middle of the second semester of grade 7.

Variable	*B*	SE	*t*	*P*	VIF
Sleep duration (h)	−0.191	0.051	−3.738 **	0.000	1.062
Sleep quality	0.125	0.053	2.335 *	0.020	1.164
Homework duration (min)	0.206	0.051	4.064 **	0.000	1.049
Exercise duration (min)	−0.093	0.050	−1.841	0.066	1.035
Self-assessment scores	0.174	0.052	3.344 **	0.001	1.104
Parental involvement scores	0.127	0.051	2.488 *	0.013	1.058
Number of daily planned tasks	−0.137	0.052	−2.616 **	0.009	1.120
Duration of electronic device use (min)	−0.067	0.053	−1.271	0.205	1.133

** Significant at *p* < 0.01. * Significant at *p* < 0.05.

**Figure A1:**
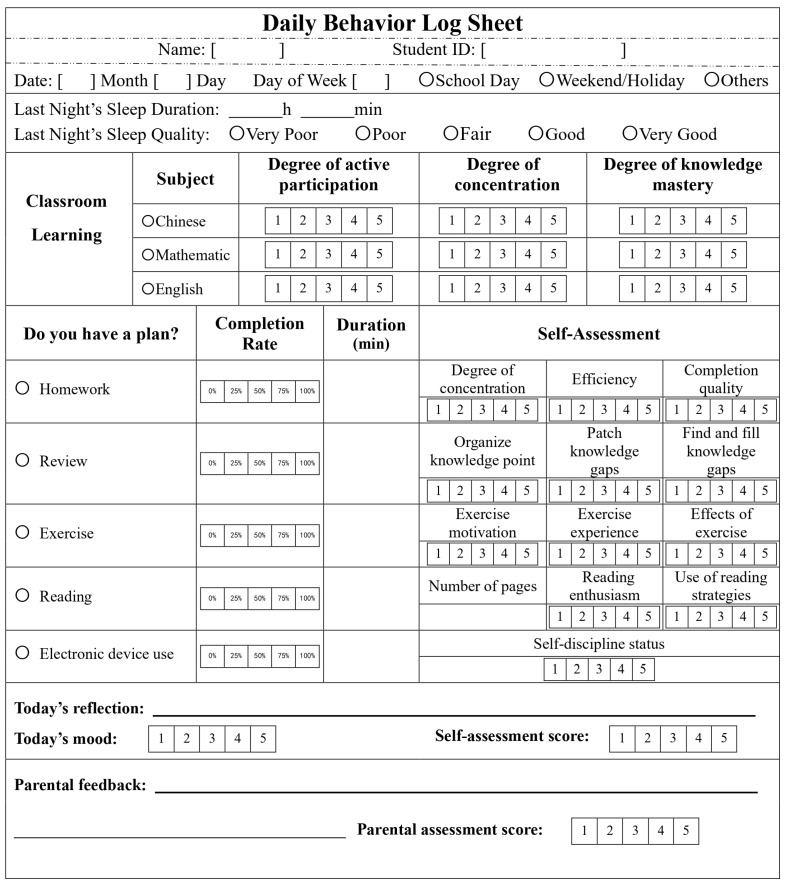
Paper-based daily behavior log sheet.

**Figure A2:**
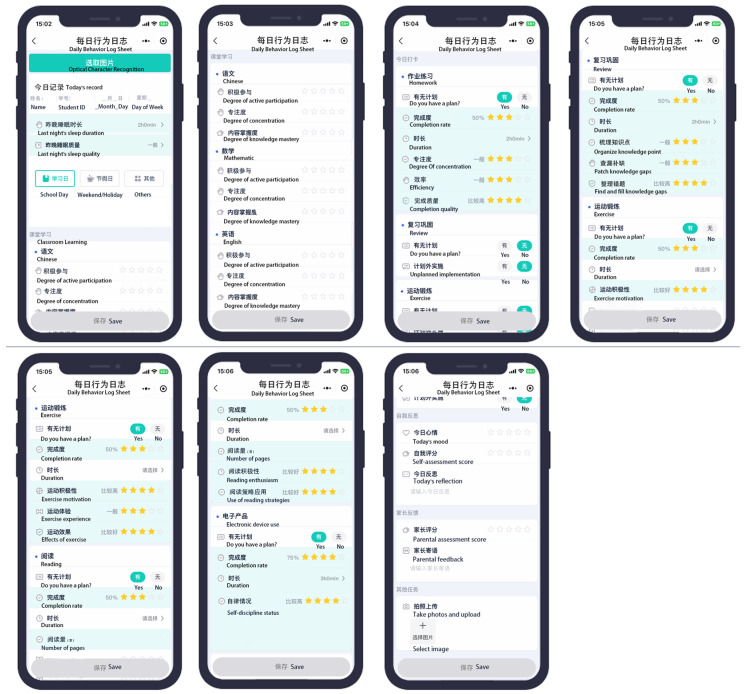
Self-management app.

## Data Availability

The data will be available in the following repository: doi: 10.17632/ygf9b7yfpd.1 (accessed on 2 January 2025).

## Appendix C

**Table A1:** Regression results for all indicators at the end of the first semester in grade 7.

Variable	*B*	SE	*t*	*P*	VIF
Sleep duration (h)	−0.206	0.051	−4.055 **	0.000	1.062
Sleep quality	0.134	0.053	2.519 *	0.012	1.164
Homework duration (min)	0.200	0.050	3.959 **	0.000	1.049
Exercise duration (min)	−0.112	0.050	−2.227 *	0.027	1.035
Self-assessment scores	0.169	0.052	3.259 **	0.001	1.104
Parental involvement scores	0.149	0.051	2.941 **	0.003	1.058
Number of daily planned tasks	−0.115	0.052	−2.210 *	0.028	1.120
Total days of log sheet recording	0.024	—	0.243	0.808	4.117
Daily task execution rate	0.008	—	0.143	0.887	1.166
Total number of classroom learning reflections	0.040	—	0.398	0.691	4.223
Review duration (min)	−0.019	—	−0.362	0.718	1.184
Duration of electronic device use (min)	−0.035	—	−0.679	0.497	1.077
Reading duration (min)	0.045	—	0.804	0.422	1.318
Parental assessment scores	0.058	—	0.945	0.345	1.529
Daily mood scores	−0.076	—	−0.960	0.338	2.567

Note: SE = standard error; VIF = variance inflation factor. ** Significant at *p* < 0.01. * Significant at *p* < 0.05.

**Table A2:** Regression results for all indicators in the middle of the second semester in grade 7.

Variable	*B*	SE	*t*	*P*	VIF
Sleep duration (h)	−0.191	0.051	−3.738 **	0.000	1.062
Sleep quality	0.125	0.053	2.335 *	0.020	1.164
Homework duration (min)	0.206	0.051	4.064 **	0.000	1.049
Exercise duration (min)	−0.093	0.050	−1.841	0.066	1.035
Self-assessment scores	0.174	0.052	3.344 **	0.001	1.104
Parental involvement scores	0.127	0.051	2.488 *	0.013	1.058
Number of daily planned tasks	−0.137	0.052	−2.616 **	0.009	1.120
Total days of log sheet recording	0.024	—	0.237	0.812	4.117
Daily task execution rate	0.040	—	0.396	0.693	4.223
Total number of classroom learning reflections	−0.014	—	−0.253	0.800	1.184
Review duration (min)	0.026	—	0.485	0.628	1.166
Duration of electronic device use (min)	−0.069	—	−0.863	0.389	2.567
Reading duration (min)	−0.040	—	−0.772	0.441	1.077
Parental assessment scores	0.070	—	1.135	0.257	1.529
Daily mood scores	0.071	—	1.244	0.214	1.318

** Significant at *p* < 0.01. * Significant at *p* < 0.05.
